# Understanding Recovery Colleges Through Partaker Narratives: Qualitative Diary-Interview Study

**DOI:** 10.2196/89886

**Published:** 2026-08-14

**Authors:** Marloes M C van Wezel, Christien Muusse, Jenny Boumans, Floris J A Scheerstra, Danny van der Spek, Judith Lize, Kelly Leunen, Dike van de Mheen, Hans Kroon

**Affiliations:** 1 Tranzo Scientific Center for Care and Wellbeing, School of Social and Behavioural Sciences, Tilburg University, Warandelaan 2, Tilburg, North Brabant, 5037AB, The Netherlands, 31 30 297 1173; 2 Department of Reintegration and Care, Trimbos Institute, Utrecht, The Netherlands; 3 Enik Recovery College, Lister, Utrecht, The Netherlands

**Keywords:** peer support, mental health challenges, experiential knowledge, mental health recovery, recovery education, recovery narratives

## Abstract

**Background:**

Recovery colleges (RCs) facilitate peer-supported learning communities where people experiencing mental disruption work on recovery. How recovery processes unfold, especially within RCs, remains insufficiently understood.

**Objective:**

Using a narrative approach, this study scrutinized how recovery processes unfold for RC partakers within RCs and in their everyday lives, and what RC attendance means in those processes.

**Methods:**

This study’s design, recruitment, data collection, analysis, and manuscript drafting were co-created with experiential researchers (ie, RC partakers). Following an interview study (N=26), a diary-interview study was conducted (N=5) between November 2022 and January 2023. RC partakers completed qualitative diaries for 1 or 2 months, every other day, and reflected on this diary period in an evaluative interview (conducted by an academic and an experiential researcher). Recovery narratives of 3 diary participants (Emma, Robin, and Norah) are presented.

**Results:**

For Emma, peer-to-peer contact within the RC helps rebuild a supportive social network and stimulates her to face her anxieties. Robin described how RC attendance offers new perspectives, invites her to trust her own judgments, and reconsider possibilities such as paid employment. Norah’s narrative illustrates how RC partaking in various capacities supported a shift in her identity from feeling like a failure to seeing herself as a valuable contributor. Considering all narratives, a cross-case analysis foregrounded the value of experiential knowledge in the recovery processes of the RC partakers. Two mutually reinforcing learning processes were identified: learning by exchanging and learning by doing. It also became clear that the RC explicitly facilitated contexts in which these processes can unfold, as partakers experienced recognition, inspiration, hope, perspective, supportive structures, and social safety. Experiences stemmed from learning processes and acted as catalysts for further learning. Importantly, these processes were not linear: partakers also experienced challenges in their everyday lives and within the RC.

**Conclusions:**

Our findings underscore the value of RCs in landscapes of mental care and support as they explicitly facilitate space for exchanging experiences among peers and experimenting with new behaviors. We illustrate the importance of investigating supportive processes and contexts to understand how recovery unfolds. We also present practical implications for RC offerings and participatory health initiatives more broadly.

## Introduction

### Background

Recovery colleges (RCs) facilitate peer-supported learning communities where people experiencing mental vulnerabilities or disruption (ie, peers) engage in their recovery process [1,2]. Recovery processes are considered “black boxes” [3], and how they unfold remains insufficiently understood. Based on a narrative approach, this study scrutinized how recovery processes of RC partakers unfold, with a specific focus on the meaning of RC attendance within their everyday lives.

### Defining Recovery as Situated, Relational Learning Processes

Defining recovery requires considering what it means to experience mental disruption (irrespective of formal diagnoses). This does not only involve intrinsic distress or “symptoms” but also stigmatizing and marginalizing interactions with individuals, organizations, communities, and systems [4,5]. Within such complex experiences, people often encounter feelings of powerlessness, hopelessness, and loss of identity and self-confidence. As Wilma Boevink described [6]:

*During my psychiatric career I got reduced to different diagnoses and became detached from my original experiences. My symptoms and the context in which they developed lost their meaningful connection. [...]. I had experiences I did not understand, which made me feel powerless*.

First-person accounts as such fueled a redefinition of recovery [7,8], moving beyond a focus on symptom reduction [9,10]. As such, recovery is defined as the personal learning processes of (re)developing identity, agency, and meaning in life [11,12]. Over the years, academics attempted to conceptualize recovery in frameworks, paradigms, and models [13-16]. Among them is the CHIME (connectedness, hope, identity, meaningfulness, and empowerment) framework [14], describing 5 recovery processes (ie, connectedness, hope, identity, meaningfulness, and empowerment).

Many conceptualizations of recovery have an intrapsychic focus, suggesting that recovery as a transformational process takes place within the individual, as a transformation of “the self.” Though, several scholars have made philosophically and empirically grounded propositions that such transformation is not merely personal or individual, but relational [17-20], as individuals are not “in relationships” but “relational beings” [21]. In terms of recovery, experiences of empowerment, hope, and identity largely exist by virtue of interactions between individuals and their relations and situated (social) contexts [22,23].

### RCs as Recovery-Supportive Learning Contexts

RCs aspire to facilitate contexts that support recovery by approaching it from an educational rather than a clinical perspective [2,24]. In that line, learning theories offer a useful lens to understand how RC contexts can facilitate a space for recovery processes to unfold [25]. Across diverse learning traditions, 3 recurring principles are particularly relevant. Learning is proposed to be (1) inherently situated and relational, (2) a transformative process through experience, and (3) driven by learners’ agency, through which learners shape their own learning trajectories (eg, [26-28]). Considering this, principles of learning and the characterization of recovery processes seem to overlap, which RCs explicitly bring into practice.

First, RCs facilitate peer-supported learning communities, foregrounding the relational and situated nature of recovery. Knowledge hierarchies and epistemic inequalities are actively dismantled [29], and instead an “ethic of dialogue” prevails [25]. Within RCs, collaborative learning takes place through the exchange of experiences among peers, where all input is equally valued. In peer interactions, individuals can learn from role models (in which they recognize themselves) enacting hopeful, inspiring behaviors [30] and give meaning to their experiences through comparison with others [31]. Like that, peers can be “authors of alternative identities,” countering self-stigmatization and increasing confidence in positive change [32].

Second, RCs support transformative learning processes. Within RCs, there are opportunities to reflect on lived experiences, explore alternative narratives, and experiment with new roles and behaviors [33]. This especially foregrounds in how RCs (and their offerings) are organized. Namely, RC practices are collaboratively shaped by all partakers [2,34] and can therefore be considered communities of practice [26]: peer communities where everyone contributes through their personal experiences.

Third, RCs emphasize the empowerment and agency of partakers, inviting them to acknowledge their expertise in their own recovery process. The diversity of RC activities and roles, combined with the absence of predefined pathways, enables partakers to acquire knowledge and skills that they personally value. Namely, partakers can shape their involvement in ways that are meaningful to them [33]. By allowing partakers to actively determine how they engage, RCs seek to redefine power dynamics, transforming peers from “passive patients” to “active co-creators” in their recovery [35,36].

### Understanding “How”

Literature suggests that RCs can successfully foster recovery, with positive effects reported for, for example, experienced empowerment, social support, and societal participation [37,38]. Scholars have also identified mechanisms of change or action that can explain these outcomes. Proposed mechanisms of change within RCs are facilitating empowering and judgment-free environments, fostering supportive relationships, promoting personal growth through collaborative learning, and redistributing power through reciprocity, co-creation, and role modeling [1,25,39-41].

While existing work provides important insights into “why” RCs can foster recovery, they offer more limited understanding of “how” such processes unfold in practice, across contexts, and over time. Especially the interplay between RC experiences and broader everyday contexts remains underexplored. Addressing this gap requires a process-oriented approach that attends to partakers’ experiences within the RC in relation to their everyday lives, including their social networks, living conditions, use of care and support services, and engagement in meaningful activities. Therefore, using a narrative approach, this study explores recovery processes of RC partakers within and beyond the RC and the role of RC attendance in how these processes unfold.

## Methods

### Design

This diary-interview study was part of a large mixed-methods, co-created investigation of the meaning and effectiveness of RCs [42]. The study was carried out by collaborative teams of academic and experiential researchers (ie, RC partakers), who were substantially involved across all stages of the research process, including study design, data collection, analysis, and dissemination of results [43,44]. Centralizing co-creation and experiential knowledge in our research aligned with RCs’ philosophy and contributed to improved research quality [45-47]. While similar approaches have been described in the literature as co-production (eg, [43]), our approach is best characterized as participatory research [44], given the challenges of realizing full shared decision-making and ownership within the context of an academic dissertation.

An analysis of participatory observations, internal documents, and interviews has been reported elsewhere [33]. This diary-interview study was a follow-up to the “initial interviews.” It combines synthesized, dialogical reflections from interviews with diary entries written in close temporal proximity to actual events. In this way, we captured both interpretations constructed in co-creation (between researcher and participant, in the interviews) and individually (by the participant, in their diary [48-50]).

### Recruitment and Participants

Interviewees from the initial interviews [33] were invited to participate in the follow-up diary-interview study during debriefing. They received a flyer with more information about the study’s goals and procedures and took time to consider their willingness to participate. Five participants were enrolled (mean age 49, SD 9.5 years, all identified as women), of whom 3 narratives are presented (Emma, Robin, and Norah).

### Setting

The study was conducted at Enik Recovery College (ie, Enik RC or Enik in short), a Dutch RC in the region of Utrecht, hosting 7 locations. Enik RC was established in 2015 and is nationally recognized as a leading example. A 100% peer-run philosophy is central: Enik RC is co-created by and for peers. Enik RC offers an elaborate self-help recovery curriculum, including established courses such as the Wellness Recovery Action Planning (WRAP [51]) and Honest, Open, Proud (HOP [52]), and co-created activities and workgroups. There are also social meeting points for peer-to-peer contact and volunteering opportunities. In light of these RC offerings, we use “RC partaker” as an overall term to indicate individuals active at the RC (in any capacity) with further specifications as RC students (attendants of workshops or activities), visitors (of the social meeting point), volunteers, and peer workers (RC employees). RC attendance is free of charge, and there are no diagnostic criteria or formal referrals. In 2024, Enik RC offered 700 curricular activities and welcomed 1300 unique students, 50 employees, and 200 volunteers [53].

### Materials

Diary themes were established in co-creation with experiential researchers. Initially, academic researchers envisioned an experience sampling method study, so in a co-design session, validated questionnaires such as the Netherlands Empowerment List [54], Mental Health Inventory-5 [55], and the Maastricht Quality of Life Scale [56] were consulted as inspiration. However, discussions within the experiential research team revealed that a qualitative, less frequent diary approach would better capture context-dependent meaning of recovery-related events and align more closely with participants’ needs. One experiential researcher, for example, shared how being asked 5 times per day, “How do you feel now?” would be very stressful, and another experiential researcher shared how they were overwhelmed by questionnaires in health care already.

Inspired by questionnaire items (eg, “I am not afraid to rely on myself” and “I do the things that I think are important” from the Netherlands Empowerment List) and personal experiences of experiential researchers, five diary themes were defined: (1) meaning and fulfillment, (2) social support, (3) recognition and acknowledgment, (4) self-image and confidence, and (5) empowerment. For each diary theme, participants answered a dichotomous question (eg, “Were you able to do things you found important during the past 24 h?” for meaning and fulfillment, with answer options “predominantly yes” vs “predominantly no”). Then they provided qualitative reflections on the theme and on whether (not) attending the RC impacted their entries.

While the diary-interview study was the dominant data source, the initial interviews of Emma, Robin, and Norah were also reanalyzed as they provided more context for their recovery stories. These interviews were semistructured and focused on first encounters with the RC, experienced RC dynamics, and experienced impacts on recovery [33].

### Procedure

The diary interview study was conducted from November 2022 to January 2023, including a start-up session, the diary period, and an evaluative interview. All start-up sessions (not analyzed) took place at the RC and served as design sessions during which participants selected predefined themes to be included in their diaries. All participants decided to include all themes.

**Figure 1.**
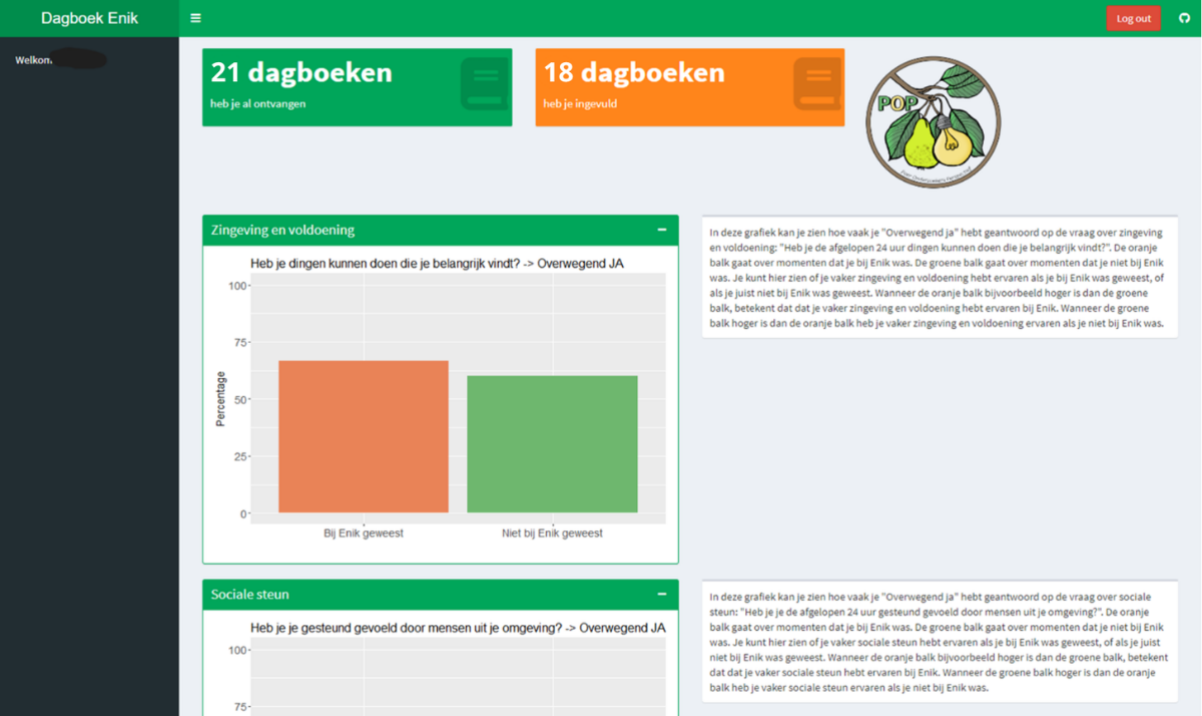
Example of a personalized Shiny dashboard.

The diary period lasted 1 month, with an option to extend it by another month (which Emma and Norah did). Diaries were distributed every other day via Qualtrics (compliance rates: Emma=48%, 15/31; Robin=81%, 13/16; and Norah=68%, 21/31). During the diary period, participants could log into a personalized RShiny dashboard displaying the number of diaries received (top left, green) and completed (top right, orange), graphs per diary theme (displaying frequency of “predominantly yes”; orange bar=days of RC attendance and green bar=days of nonattendance), and explanatory text balloons (Figure 1).

Aligned with the initial interviews, the evaluative interviews were conducted by an academic and an experiential researcher, at the RC or the participant’s home. Both interviewers had a shared and equivalent role in asking questions. While one interviewer typically opened the conversation, follow-up questions emerged naturally through the dialogue. During the evaluative interviews, participants logged into their dashboard and gave meaning to the graphs, discussing important events during the diary period. Interviewers systematically invited participants to relate salient patterns in the graphs to their lived experiences, for instance, by asking about typical days associated with higher or lower scores or how participants understood observed differences between RC and non-RC days. Qualitative diary entries were analyzed afterward and were not accessed prior to or during this evaluative interview.

### Data Analysis

Data analysis was collaborative and co-created. To integrate the diverse perspectives, knowledge, and competencies of the research team [45,57], the analysis was open and flexible [33]. Theoretically, elements of narrative approaches and reflexive thematic analysis informed our analysis strategy. Narrative approaches explore the intertwinement of processes and their situated meaning in everyday life [58], considering not only content but also narrative form [59]. Furthermore, the diversity of perspectives and knowledge within the research group is acknowledged as a valuable source in interpretative and reflexive analysis [60,61]. Our collaborative analysis was iterative and combined deductive and inductive analytic strategies. The analytic team (4 experiential researchers and the first author) met in 6 co-analysis sessions.

In the first session, we sharpened our research question, developed an analytic plan, and divided the transcripts to familiarize ourselves with the data (the first author read all transcripts). We began with the evaluative interview data, expecting these to highlight the most prominent events and plot twists from the diary period. In the second session, we explored the evaluative interviews deductively using the predefined diary themes. For each theme, we explored temporal development, influencing factors (general and RC-specific), and their meaning within recovery processes. These factors were identified inductively and organized with colored sticky notes to establish a thematic framework. We then noted recurring and unique patterns across participants’ accounts. In session 3, we attempted to compile composite narratives (inspired by Willis [62]) using the diaries, synthesizing themes and summarizing profiles across participants. Soon, we realized that such an approach risked oversimplifying and decontextualizing complex, unique recovery narratives [59].

To preserve the richness and situated meaning of participants’ experiences, we therefore selected Emma, Robin, and Norah for in-depth analysis, focusing on how meaning unfolded within their lived contexts [58]. These cases were selected based on data richness and the diversity of their recovery processes and RC involvement (including the duration and nature of attendance). A shared narrative structure was developed to explore participants’ recovery journeys, including contextual backgrounds, personal meanings of recovery, and experiences during the diary period, with particular attention to RC-specific processes unfolding. In preparation for sessions 4 and 5, we reread all data from the 3 selected participants with this structure in mind. The interviews provided overarching reflections, while the diaries offered more detailed insights into temporal development and concrete, everyday examples of recovery-related experiences. We also checked for discrepancies between diary entries and retrospective reflections, which did not occur. Sessions 4 and 5 served to inductively code important themes per participant. Based on this, the first author wrote concept narratives (including reflections) in Dutch and fine-tuned them within the research team and in correspondence with the participants. In a sixth session, we explored cross-case patterns across the 3 narratives and identified overarching processes that captured how interaction within Enik RC contributed to recovery. To achieve this, we examined how cross-case patterns related and interacted and explored whether patterns could be meaningfully organized using frameworks such as self-determination theory and CHIME. Through iterative comparison and reclustering, we ultimately identified 2 overarching processes that best captured the observed dynamics. This analysis was fine-tuned in collaboration during paper drafting and revision. Coauthors (academic and experiential researchers) provided feedback on paper drafts, which the first author incorporated.

Consensus within the co-analysis team was reached through iterative dialogues. The first author facilitated the process by guiding discussions, summarizing interpretations, and drafting analytical texts between sessions. To support a diversity of perspectives during co-analysis, we intentionally had an experiential researcher take notes on the flip-over during our sessions, and we routinely brought preliminary interpretations back to the group for critical reflection and refinement. While consensus was typically achieved in these dialogues, final responsibility for analytic decisions formally rested with the first author.

### Ethical Considerations

Experiential researchers emphasized ownership of data collection and analysis by (1) inviting participants to partly shape their diaries, (2) scheduling evaluative interviews to jointly interpret diary scores, and (3) developing a personalized dashboard that participants could bring to these interviews as input.

All participants provided written informed consent. For member checks, Emma, Robin, and Norah were contacted via email. They were invited to read a first concept of their narratives and provided additional comments. Names and contextual details (eg, family relations) have been altered in collaboration with the participants to achieve the desired level of anonymity [63,64]. Participants indicated to what extent they wanted to disguise their identity and which details they considered identifiable. Requested revisions were made by the first author based on participants’ input and subsequently returned for confirmation prior to publication. Ethical approval was granted by the Ethical Review Board of Tilburg University (TSB_RP390).

### Reflexivity

The work was part of the first author’s PhD dissertation, who also had lived experience with recovery. Furthermore, academic researchers with scientific knowledge of recovery and mental health, and experiential researchers, who were RC partakers, collaborated (see a study by van Wezel et al [33] for details on their backgrounds). Within the analysis, several layers were intertwined: (1) participants’ analytical reflections on their diary period from the evaluative interviews, (2) researchers’ analytical reflections (shaped by their own experiences), and (3) theoretical frameworks and predefined themes. Throughout the analysis, we critically reflected on where insights stemmed from.

We also reflected on how varying degrees of contextual access shaped the analysis. Some analysts only had access to written transcripts, while others had conducted interviews, and in Emma’s case, even visited her home. This provided additional insights into narrative form and context that might be unavailable in written text. To address this, interviewers shared their observations about tone and setting, ensuring that such contextual nuances informed the interpretation [59].

Moreover, the co-research team stemming from the same community as the research participants prompted critical reflections regarding internal confidentiality [64]. While names were omitted from the data, original contextual details were retained during analysis. Experiential researchers sometimes believed they identified participants, potentially introducing assumptions based on prior knowledge. In reflexive dialogues, we discussed whether our interpretations were grounded in the data or influenced by personal assumptions. We also considered how speculating about participants’ identities—often an attempt to enrich our understanding—carried analytic risks, as it may lead to unfounded conclusions.

## Results

### Overview

This section first provides the narratives of 3 RC partakers: Emma, Robin, and Norah (descriptives in Table 1). The narratives provide insights into what recovery means to them and how their recovery processes unfold within everyday life and the RC. Notably, all narratives stem from participants with sustained engagement in the RC, and they predominantly emphasize experiences of growth. As an analytic structure, each narrative concludes with a short reflection, highlighting which processes are most prominent and how the RC plays a role in them. Finally, a cross-case analysis is presented, synthesizing insights from the narratives and identifying common threads.

**Table 1.** Descriptive information of Emma, Robin, and Norah.

Pseudonym	Age category (years)	Gender	RC^a^ partaker roles	Duration of RC involvement (prestudy; years)
Emma	50‐59	Female	RC student, visitor, volunteer	3‐4
Robin	40‐49	Female	RC student, visitor	1‐2
Norah	50‐59	Female	RC peer worker (former volunteer, student, visitor)	≥4

a RC: recovery college.

### Emma: Redefining Social Contact

Emma first contacted us by email, responding to the recruitment for our survey study. Her email was critical. She warned of selection bias, reaching only those who benefit from the RC, as those who do not tend to drop out. We invited her to discuss this further and pointed at the interviews. She wanted to participate. We met Emma for the first time at her apartment, just outside Utrecht. Due to physical impairments she experienced at that time, she was unable to travel to the RC. After this initial interview, Emma decided to participate in the diary-interview study. We concluded the 2-month diary period with a second interview at her home. Recalling the diary period, Emma remarked that she often forgot to complete diaries when she felt unwell:

*Then I’m really stuck in my head and stressed out, or anxious. I don’t really have room for anything else anymore. I get so caught up in my thoughts and I don’t even think about it [completing the diary, red.].*

We decided to keep this in mind when analyzing her dashboard.

In our initial interview, Emma shared that social contact induces a lot of tension. In the past, she had been bullied, and she had experienced an unsafe childhood. Paralyzing anxiety and depressive feelings regularly took hold of her, and her self-confidence was low. Besides, she did not have a supportive social network for quite some time:

*I moved here a few years ago and I had nothing. And it was really difficult to meet people, also because I am not easily comfortable, especially in the past. [...] I always feel like an odd one out and that I have to prove myself more. That I can’t truly be myself. And that they disapprove that I have welfare benefits and do not work*.

She wanted to make a difference for others and had tried volunteering at several places, but “that just didn’t work.” For Emma, mentalization-based treatment therapy was the start of a learning process to reevaluate social contact:

*That is where the roots are. You learn that people think of you differently from what you think. Because honestly, I felt utterly rejected by everyone, and I started checking that. Often, it simply turned out to be incorrect.*

She became active at the RC as a hostess, which helped her to overcome years of loneliness and build a supportive social network:

*For a long time I just sat at home, hidden from everything and everyone. [...] I met my best friends there [at the RC, red.].*

Contrary to her earlier beliefs, Emma now finds that social contact, if with the right people, brings her energy and happiness. As she wrote in her diary:

*I told my partner: I feel happy. I have a home, two bunnies, a partner, three friends, and Enik !! Because of Enik I more and more experience that I like to be surrounded by people. Something that I didn’t have before. Then, I was really lonely.*

In her expanding social network, she finds support, both emotionally and practically. For example, when she experienced internet problems, which she found stressful, she wrote in her diary:

*All kinds of calamities come to mind. I can’t defend myself against them. Terrifying*.

She talks about it with her friends, her support worker, and therapist, and 1 day later, she writes:

*I need assurance, putting things into perspective, and practical advice. And I receive that. So happy with my circle!!! [...] My anxieties about everything that could go wrong took hold of me. But I reached out about it to my environment. It is scary. But there is support*.

Besides volunteering at the social meeting point, Emma takes part in courses and activities at the RC. Soon she developed ideas for new activities, which she put into practice and facilitated. She also facilitated other existing courses. She considers the RC as a safe haven she can always return to, to practice what she learns in therapy and services for mental health support.

All in all, Emma feels she has grown in showing her true self. She shared in our initial interview:

*I have become much more visible [...]. When I enter, I am here. I am not a grey little mouse that sneaks in and says nothing. In the past, I did not have a conversation [...]. Now, I say a lot*.

During the diary period, Emma practiced further with self-expression. She wrote:

*I went to Enik and attended a workgroup. I felt very bad about myself there, because I talked myself down and felt that I couldn’t make anything of it. At the end I said I didn’t like it at all for that reason. And then I received some nice reactions from those who facilitated it. I felt sad, but that was OK. After that, I talked to the JijenIk’er [hostess, red.] in the social meeting point. It was very nice to find so much recognition with each other. I left with a smile on my face. That was seen by [peer worker] who said something about it, and I liked that a lot, that I was seen*.

A week later she wrote:

*I went to the workgroup at Enik. I was really dreading it because I was so severely critical of my own writing. But it was very good that I went because the theme was “being allowed to make mistakes”. And I also just discussed that with [mental healthcare provider] that morning. Because I expressed my uncertainties last time, a connection and safety for me emerged in the group, and because of that, others expressed uncertainties too. So it was very nice to practice in a safe environment. [...] And I dared to read my writing aloud after all. [...] The more I open up, the more I receive in the form of feeling seen, that I am taken into account, that people appreciate me... That is a really nice feeling, and it makes me more proactive*.

However, when Emma experienced stress due to her internet problems, she experienced crossing her boundaries when sharing something in the workgroup. While she was happy she self-expressed a few weeks before, she now wrote:

*Despite that I wanted to leave, I stayed. [...] Read my pieces where I made myself far too vulnerable in front of a group where I did not feel safe*.

Emma not only practices within the RC but also in other contexts. She shared in the evaluative interview how she used to experience complicated interactions with a street vendor:

*There is always a street vendor, and he always looked at me very angry, and did not say anything when I greeted him. Every time I felt so miserable about that. While it is someone I don’t know at all, [...] but the theme of “rejection” surfaced. And then I discussed this in the workgroup and the conclusion was: “Go there and ask him: Are you angry with me?” [...] Eventually, I went to the man, and I asked whether we could have a chat. And then we cleared the air, and now we greet each other friendly. So, I would have never done that without the workgroup!*

In this situation, Emma faced her social anxiety and experienced success in that. Emma explained how the workgroup’s facilitator played a crucial role in this and concluded how he served as an inspirational example:

*If my partner had said, “Go and have a chat with that vendor”, I am not sure whether I’d have done it. [...] If he [the facilitator] says something, I take that very seriously. [...] I find him a very socially intelligent man. He also says we are very similar in the things we struggle with. But he is more advanced in it*.

Due to her autism diagnosis she received a few years ago, and the support that came with it, Emma continues to learn what she needs:

*Through the discovery of that autism, I suddenly realized, “I have to take good care of myself. How do I do that?*”

Conversations with her psychologist are helpful, as she wrote in her diary:

*Because there I can have good, supportive, insightful conversations !!*

From this connection with herself and insights into what she needs, Emma increasingly practices setting boundaries. She experiences the RC as a place that stimulates this. Once, she walked away from a conversation in the social meeting point because someone was very hyperactive, and she wanted to avoid becoming overstimulated:

***Interviewer:*** *How was that for you, to take that step and stand behind the bar?*

***Emma:** It felt really good. At Enik, you learn much more to stand up for yourself anyway, to say it when you don’t like something, and that is, in fact, rewarded. [...] I’ve really learned a lot.*

Though, setting boundaries is not always successful. During the diary period, Emma was assigned a new support worker at her own request. While she was happy with this decision, it also meant a period of uncertainty, getting acquainted and attuned. Setting boundaries in this was more challenging. She wrote in her diary:

*I did not set my boundary with my support worker. The conversation took too long. I was exhausted.*

### Reflection on Emma's Narrative

Volunteering at the RC marks a key turning point for Emma. It ends years of loneliness and allows her to build a supportive social network, practice social skills, and apply what she learned (and continues to learn) in therapy and support. For Emma, the RC is a space for practice and for contact with peers. Support services offer practical support, and therapy is for acquiring insights.

Emma’s motivation to learn and develop herself is put into action when she experiences social safety. At the RC, recognition and supportive interactions when she shares vulnerabilities stimulate others to do the same, which increases safety and encourages more openness. This transition from concealment to being seen and heard, and ultimately revealing and expressing herself, increases her self-confidence.

Recognition Emma experiences within the RC also motivates her to face fears beyond its walls, further boosting self-confidence. These self-reinforcing processes are apparent throughout Emma’s narrative and fueled by experiencing new things. Despite this, Emma’s narrative also reveals that recovery is erratic, with good and more challenging days. This makes clear that the self-reinforcing cycle also works the other way around. External stressors (eg, internet problems) can disrupt her sense of safety, increasing anxiety and self-doubt: even within the RC setting.

### Robin: New Perspectives

Robin applied for the interview study after encountering the recruitment poster at the RC and requested the questions in advance so she could reflect on them. We spoke with her twice at the RC, and she completed the diary for a month. In the evaluative interview, Robin shared how journaling gave her a better overview:

*I have very little overview. And by filling out the diaries, you acquire that. You realize that it [attending the RC, red.] offers you more than you thought. I appreciated that a lot*.

Journaling also led her to redefine what she considers meaningful activities, such as cleaning the house and cooking dinner for her family.

She recounted how she came into contact with the RC by coincidence, during her role as a moderator of peer support groups for an association that rents RC course rooms for its meetings. During that time, she felt hopeless and discouraged. For years, she had experienced depression, anxiety, and chronic fatigue:

*I was very angry that I did not have that much energy and could not enjoy things anymore. [...] I received regular care, but then not again for years. At that time, I did nothing, merely survived. [...] When I first attended [the RC, red.] I had no more hope. I remember that one of the support pillars of the WRAP was hope, and that I wanted to throw something across the room. That I thought, “Get lost with your hope, what hope?”. Then you go through all those stupid clichés. The light at the end of the tunnel. I thought, “I don’t have light at the end of the tunnel for ten years, I move on because I have to*”.

Her partner stimulated her to move from fighting it to acceptance.

*It helped that my partner said many times, “You spend energy you don’t have on something you can’t change. Just look at what is possible*.”

She was also stimulated by the RC’s offerings presented on the flyers that she distributed at the peer support meetings she moderated. As a result, she started attending RC activities: the HOP, the WRAP, and various recovery workgroups.

Besides her partner, Robin has a few good friends with whom she regularly calls or meets up. She has no lack of social contact but still experiences loneliness in how she feels. This loneliness particularly surfaces when she experiences no or little recognition from others. On such a day, she answered the “recognition” diary question as follows:

*Only my partner, because he is tired too. The rest of the world just keeps going, so it seems. It makes you feel lonely*.

Robin finds recognition with “people for whom life also did not unfold as expected or aspired,” such as peers she encounters in group therapy. She wrote in her diary:

*Someone recognized being so tired [...] that you can’t get anything done. She was very happy with that, too, that recognition, it creates a bond. [...] Contact with peers makes the difference. I feel heard and seen. And really understood, because they know what it feels like to feel this way*.

Exchanging experiences with peers occurs in various ways in Robin’s life: in group therapy, at association meetings, and in RC workgroups. Whereas group therapy and the association meetings are mostly about recognizing similar challenges (eg, fatigue or depression), peer contact within the RC is more diverse. Exchanging with peers who have lived experience with mental disruption in the broader sense helps Robin to put things into perspective. She described a moment in an RC workgroup (both during the initial and the evaluative interviews):

I was in the workgroup, and then there was someone who heard voices all day. You saw how he was suffering: it was so sad. *Becomes emotional* [...] Because sometimes I find myself very pathetic, because my friends are employed [...] and go on fun holidays. And then I saw him and thought, “Robin, stop whining, look at him. He also persists”. I find that so inspiring. You see people who suffer much more than you do. [...] It opens the possibility of seeing: What do I have? I have a home, a relationship, a child.

Robin discussed this moment with her psychologist:

*And I remember my psychologist saying, “You shouldn’t go there, this is too heavy for you.” Then I think, “Hey, listen to me, this is actually really helpful*.”

When she explained this to her psychologist, they kept insisting:

*They did not listen because they think they know what’s good for me. [...] “This is too heavy for you, but you don’t see it.” [...] And then I’m giving up, and think “Never mind.*”

Instead of listening to her psychologist, Robin keeps attending the RC. She feels that this is good for her, and this only became clearer when she completed her diaries:

*I knew it mattered, but I did not know it mattered so much for me sometimes [...] I realize that I am doing things that are good for myself, [...] that really help me move forward.*

For Robin, it confirmed that she was able to make good decisions, which increased her self-confidence. This feeling continued after she visited the RC, as she explained:

*Then I feel more powerful. On Wednesdays, I am babysitting and [...] I noticed that decisions I made, [...] all is more clear. [...] That you feel good about yourself. More confident. Usually I find myself endlessly ruminating, “Is this a good plan, or would something different be better?”. And now you think, “This is a good idea, let’s go.*”

But Robin often struggled to trust her choices. Her physical and mental complaints then seemed to take over control. On such a day, she wrote in her diary:

*I barely succeeded in doing things I find important because I was too tired and disorganized. [...] On days like that, I feel like I have too much on my plate and I don’t know anything and can’t handle anything. It is an unpleasant feeling, like life is not safe, and it happens to you and takes you by surprise. It is heavy and sad. It discourages me*.

In general, she found the question “Are you in charge of your own life?” very confronting and unpleasant to answer, as she explained in the evaluative interview:

*You can’t do anything with that. It is really in your face, you are responsible for everything yourself. [...] While at the same time you feel, “I just have to do deal with all the limitations I have.”*

For Robin, the RC is a place where she finds tools to maintain control over her life, as far as that is possible. After a day of feeling overwhelmed, she reflected in her diary on the fact that she had not attended the RC and how this could have impacted her day:

*At Enik I would have experienced more recognition. And reminders, such as: pay attention to your boundaries, what is good self-care, mind your WRAP.*

Preferably, she would attend the RC every day, but that is not possible due to her limited energy levels. Anyway, it is a place where she wants to develop further.

Besides feeling hopeless, Robin also experienced hope again. Before, she saw limited future perspectives:

*When you are depressed you think, [...] we all die someday and whatever.*

Now, she feels differently:

*Something is happening, and that is more than what has happened in the past ten years.*

She is considering paid employment again:

*In the past, when someone asked me, “Can you ever work again?,” I always said, “Yes,” but I thought “No.” Because I thought, “I say that, because I want that, but I don’t believe in it anymore.” And after a year and a half of Enik, I think, “Yes, it could be possible.” [...] Because at Enik, you see the living proof that other people who also experience(d) disruption lead a group, work there. [...] Imagine being able to work for four mornings per week; that would be fantastic. [...] You can also start with three times one hour. I think you see more possibilities here*.

She would like to work at the RC as a (voluntary) facilitator. However, she encounters obstacles in that:

*I would love to be co-facilitator, but it is unclear how this is arranged. I asked someday, but one said, “You should have your own subject and submit that” [...] but I found that very scary. [...] And I said, “Are there no groups where you can join?” But this one person said “No.” [...] And the other said, “Maybe”. And yet another person said, “There is the training ‘Learning to facilitate’.” But that seems to be some kind of secret group that is hard to join. [...] And they facilitate that three days in a row, that doesn’t work. [...] I’m already wiped out by day two.*

This touches old pain related to feeling excluded and also fosters self-doubt:

*I start making assumptions about everything. [...] Most of all I would like to know, is there anyone who has decided, “This Robin is not suitable”?*

She wants to work as an RC facilitator to make a difference for others. This is also possible as student, but as said, Robin also experiences a strong (rediscovered) desire to work. She shared:

*Imagine that Enik would cease to exist tomorrow, which I would find a real shame, but still, I would take that with me. I thought I could not be switched back “on” by anything, but I can*.

Robin was not working as a facilitator yet. In the initial interview, she said she gave up on this:

*It is sad, but it is such a pattern. There are many things that I wanted that don’t work out.*

During the diary period, becoming a facilitator no longer seemed to be a topic.

During a member check (December 2025, 2 years later), Robin requested an update to her narrative. Eventually, she became a voluntary facilitator within the RC, after a peer worker invited her to do so. This brought her a lot. At this moment, she was doing so well that she was employed for a few hours per week elsewhere. She felt “endlessly grateful” for the life she lives, which seemed impossible 5 years ago. She stated that this was achieved partly due to the RC.

### Reflection on Robin's Narrative

Robin already found support and recognition with peers in group therapy and association meetings. However, becoming acquainted with the RC opened a new pathway in her recovery process and allowed her to move forward. At the RC, Robin hears diverse experiences that offer her new perspectives. Noticing others’ struggles makes her more aware of what she cherishes in life. She learns to trust her own choices and keeps attending the RC, despite her psychologist’s advice, because she finds it helpful and experiences change that she had not experienced for a long time. Besides hopelessness, hope appears, motivating her to learn, develop, and consider paid employment again: something she once thought impossible.

Nevertheless, Robin’s recovery process is not a linear, upward process. She often feels out of control and dominated by her complaints. The struggle of becoming an RC facilitator also evokes pain and self-doubt. Though, when we inquire about the impact of these struggles, Robin emphasizes that these do not temper her enthusiasm for the RC. There, she found a self-chosen place where she experiences that her recovery is not “finished” yet, but advancing.

### Norah: Role Development

For us researchers, Norah is a familiar face within the RC. We frequently encounter her in the social meeting point or as a facilitator. She is enthusiastic about our research and supports us, for example, when recruiting participants, and also values participating herself. We interviewed her twice at different RC locations, and she completed a diary for 2 months. At first, she enjoyed completing the diaries, but over time, she became less elaborate, partly due to being tired after long working days:

*On the days I don’t work, I literally and figuratively have more space for it [completing the diaries, red.]*.

**Figure 2.**
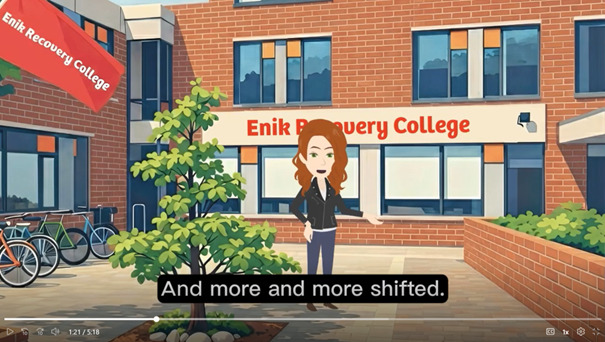
Still from Norah’s animated narrative.

To disseminate our findings, Norah accepted the invitation to translate her narrative into an animation (Multimedia Appendix 1; Figure 2).

For a long time, Norah’s life revolved around health care. She explained:

*It felt like I had missed the boat when it came to finishing education, having a relationship, kids. Those things just didn’t seem possible for me. [...] I just envisioned myself as a complete failure with no hope*.

The mental health care organization where she was “in treatment” offered a VERS (Vaardigheden emotieregulatiestoornissen; [Skills for Emotion Regulation Disorders]) training, which was the first thing she had completed in years:

*That was really good for my self-confidence*.

Around that time, she also attended the WRAP, facilitated by the organization from which she received mental health support. There, the aspiration to study or seek employment surfaced again:

*Because before I did that, I pictured myself spending the rest of my life just sitting at home, doing nothing. [...] That had to do with my self-image, not my talents, but I experienced that differently at that time*.

When she attended a lecture by an expert-by-experience about the ideology of RCs, she felt inspired:

*That was the first time I thought, “Perhaps there is still something more in store for me*.”

She started attending the RC, and she narrated about those early days:

*I found it very inspiring to meet all kinds of people who had also been through a lot of “shit,” but still found their place in life. So that gave me hope. In the sense that, I could possibly do that too. And then I just literally got back to work there. Partly through those trainings, but also through volunteering, I really noticed that I actually have quite a lot to offer. So, in a year and a half, I've gone from thinking, “I’ll be lucky if I can volunteer somewhere,” to thinking, “They’ll be very lucky to have me as a volunteer.” Of course, that is already a major thing. And then, slowly, the hope emerged: maybe I can get a job here.*

After a journey of trial and error as a student and volunteer, that is what happened. After years, she was hired as a peer worker at the RC.

The RC played a significant role in Norah’s life, as seen in her diaries. In the evaluative interview, she shared:

*For me, it reflects how important my work is for me. But also, being at Enik.*

She recounted a period where she stopped attending the RC and then became active again:

*I noticed how beneficial it was for me when I started reintegrating at Enik, just to be there again. And that it really was my place. And that I had really missed it.*

On days she was not at the RC, she was less active and had little social contact:

*Outside Enik, I simply encounter way fewer people, and spend much more time alone.*

At those moments, she felt less well, as she wrote on such a day:

*Struggling to take good care of myself in the sense of healthy food and that makes me feel sad and kind of powerless and feeling like a failure. [...] I just can’t manage to turn things around.*

Norah emphasized that social contact was very important to her. She found this at her RC job, but she was also expanding her social network elsewhere, to her own surprise:

*I’m starting to get girlfriends. I’ve never had that before. [...] The last few months, I met some nice people at Enik and at my volunteering job [not at the RC, red.], and something is starting... [...] but girlfriends, in plural. It’s really special.*

Still, the RC seemed the most significant base site in her everyday life. This became clear when there were insecurities at work during the diary period, causing a lot of stress. Norah did not complete diaries for a week, and then she wrote:

*Too sad because of the events at work last week to really achieve anything. [...] Once again, I feel very much like a failure and falling short when it comes to dealing with misunderstandings and disappointments in a mature way, and I have little confidence that I will ever really be able to do so. [...] Then thoughts that I’d be better off not being here quickly resurface.*

After this week, the insecurities proved to be less pronounced than anticipated. Norah felt supported by colleagues, friends, and family, and things were said that made her feel seen and acknowledged. She felt better again:

*I feel more powerful and I’m starting to believe in myself again. [...] The fact that I feel reconnected to my colleagues, that I enjoy my work so much, and that I’ve met new people, are important reasons for that.*

Norah explained how her job benefited her by providing weekly structure that supported good self-care. Her clear role at the RC offered safety to practice things she found scary, such as working in groups. Financially, the job allowed her to earn her own money and become less dependent on the *Uitvoeringsinstituut werknemersverzekeringen* (UWV [Dutch Employee Insurance Agency]; ie, responsible for implementing unemployment benefits). Norah remarked that these could be benefits of any employment, provided the workplace offers a supportive environment where expectations are clear and she feels connected to colleagues.

Nevertheless, the RC also offered a specific supportive context, in which she could work on her learning goals. One important learning goal for Norah was asking for support. She struggled with this, but she felt the need for it:

*I very much tend to do everything myself, and I’ve fallen flat on my face a few times because of that.*

It was an important theme in sessions with her coach, and she increasingly practiced it. This was easier when she experienced supportive social contact. For example, she more easily asked for support if others had spontaneously offered support to her before. This occurred at the RC, according to Norah, following from conscious attention and sensitivity:

*At Enik, there are a lot of very kind people, often also very sensitive people attentive to these things*.

She characterized her new friends in the same way. She also shared that asking for support at the RC was facilitated because the RC was based on shared responsibility:

*The other day, I had overloaded myself with tasks that I took over. And then [colleague] said: “Then we have to go back to the drawing board. [...] Than we have to find something for...” – That feels very much united.*

She also emphasized the impact of shared experiences with struggle, which are a given within the RC:

*Of course, here at Enik, you discuss what you struggle with a lot. [...] Any way, the safety of not having to talk about your problems, but still knowing that everyone understands some of it, or at least doesn’t disapprove it, that offers so much air and relaxation to just be who you are.*

Norah’s peer worker role helps her rebuild a positive self-image and confidence. It allows her to reciprocate experiences she had as a student and volunteer. For example, it meant a lot to her that others saw her potential:

*When I just started volunteering here, how proud I was, how I felt seen and valued, when I was asked to do a chore or brainstorm about something together. [...] Like, “People see something in me, after all”. And at that moment, I saw little to nothing in myself. [...] That I received that from others was something for my personal development... [...] I felt like, “I am up for paid employment”. That took three years, but eventually, I succeeded. And that is what I wish for others, too. To regain that self-worth and respect, and feel seen*.

She shared how her work offered her joy and meaning as she could connect people. She addressed their talents and skills and involved them in projects. As an illustration, she described a large project that she co-created with the community, and what this meant for her:

*Then I really feel empowered, thinking, “I initiated this and eventually, it works out.” It becomes what I envisioned. And people are as excited about it as I hoped. Because, of course, that is what it’s all about in the end. It’s not about me wanting this project so much, it’s about those people going home, feeling inspired.*

### Reflection on Norah's Narrative

Norah describes her development across various roles within the RC. Even as a peer worker, her personal growth continues. She still struggles with low self-image and wants to learn to ask for support when needed. Beyond support from a coach, the RC is important: paid employment offers structure, social contact, and financial independence, while the RC facilitates a context she finds recovery supportive. She highlights the special bond among RC partakers based on shared experiences with mental vulnerability and unconditional support. As a peer worker, she learns to reciprocate what she once experienced: being seen, valued, and encouraged. She learns how she is of value to others, growing her self-confidence.

It becomes evident that the RC encompasses a significant part of her everyday life, and outside it, loneliness often prevails. She struggles with self-motivation and self-worth. While Norah experiences great value in her RC involvement, its central role can also introduce vulnerability: when the RC is both workplace and support system, work-related stressors can threaten her sense of a “safe haven.” However, during the diary period, Norah also builds a supportive network outside the RC.

### Cross-Case Analysis

#### Overview

Within the RC, Emma, Robin, and Norah experienced recognition, inspiration, hope, supportive structures, and social safety. These were not discrete outcomes, but ongoing, intertwined, and situated processes. Peer interactions especially surfaced as meaningful contexts for these experiences. Similar experiences sometimes arose in partakers’ everyday lives, for example, when experiencing support and safety in relationships with significant others or care and support providers. Nonetheless, the RC deliberately facilitated space for the exchange and development of experiential knowledge, which could foster recovery processes to unfold.

#### Learning by Exchanging and Learning by Doing

Across cases, we identified 2 unfolding processes: learning by exchanging and learning by doing. While analytically distinct, these were intertwined and mutually reinforcing. Learning by exchange was primarily a process of knowledge expansion, involving interpersonal and relational sense-making through (non)verbal input, such as insights, role modeling, or supportive behaviors. Emma, Robin, and Norah all noted peers in whom they recognized aspects of themselves, motivating identity reconstruction and new behaviors. For example, Emma’s exchange with a peer facilitator stimulated her to overcome her social anxiety, Robin reconnected with her ambition to work, and Norah, experiencing collegial support, became more aware of seeking help.

Learning by doing transformed abstract knowledge that was previously detached from the self into lived experiential knowledge through practice. Emma not only “knew” social interactions are not as daunting as she used to believe, Robin not only “knew” that she can make good decisions for herself, and Norah not only “knew” that she has things to offer: after “doing,” they “experienced” it too.

#### Challenges

The narratives also revealed experienced challenges. Recovery was nonlinear and erratic, with partakers facing negative self-cognitions and emotions. In addition, navigating the RC could be challenging, for example, when implicit power relations and role tensions foregrounded. Norah’s narrative illustrated the fragility of an RC functioning both as a workplace and a safe haven, given its central role in her everyday life. Robin encountered unclear pathways to becoming a facilitator: a role that only became accessible upon invitation. These examples highlight how implicit power dynamics within RC context could shape and challenge attendance.

## Discussion

### Principal Findings

Through detailed, narrative accounts of 3 RC partakers, we have acquired more insights into how their recovery processes unfolded within the RC and their everyday lives. Our findings showed that recovery of the selected RC partakers encompassed processes of learning by exchanging and learning by doing. The RC context was explicitly designed for these processes to evolve, as the peer-supported learning community facilitated space for exchanging experiences among peers and for practicing and experimenting with new behaviors. As such, the RC could be understood as a “community of practice” [26].

The narratives especially foregrounded how the RC partakers valued lived experiences and experiential knowledge as sources to learn from. As Moran et al [65] described:

*The use of one’s lived experience as a source of knowledge transforms that which is most stigmatized into an asset.*

Our findings help to understand the potential value of experiential knowledge in recovery processes [66,67] and align with lived experience accounts articulating experiential knowledge, education, peer support, and personal agency as vital aspects of navigating recovery journeys [12,23].

Existing literature has elaborated on RC experiences and perceived impacts, making clear what RCs potentially have to offer [38,39,68]. RCs are proposed as empowering contexts that redistribute power and foster supportive relationships and personal growth [1,25,39-41]. Our previous work described how RC partakers experienced the RC as spaces for learning, social contact, and low-threshold, co-created organizational forms, inviting to experiment with new roles and skills [33]. Through a narrative approach, this study has acquired more enriched insights into how these spaces can be meaningful contexts for recovery processes to unfold.

Nonetheless, the narratives also illustrated the ongoing and erratic nature of recovery, as RC partakers encountered (mental health) challenges alongside trajectories of growth. Importantly, some challenges specifically emerged within the RC context: Emma suddenly feeling unsafe in the workgroup, Robin feeling rejected in her ambitions to become a facilitator, and Norah experiencing work-related, disruptive tensions. These accounts corroborate previous studies reporting that RC attendance is not necessarily easy or straightforward and may evoke tensions or negative experiences too [33,69,70]. These included, for example, challenging power dynamics (eg, in the context of decision-making) and role tensions, which have also been described in contexts of peer-led mental health care [71] and participatory research [72].

Altogether, our findings suggest that RCs should not be understood as “causing” recovery, but as facilitating conditions under which recovery may become possible at certain moments. This resonates with the concept of “generative causation” as described in realist evaluation literature [73,74]. As such, recovery unfolded over time through interwoven, personal, relational, and situated processes, echoing broader understandings of recovery as nonlinear, contextually embedded, and unique [17,23,75].

### Theoretical Implications

Unraveling how recovery can be fostered often involves a search for mechanisms of action or change, and scholars have identified such mechanisms in peer-supported and recovery educational contexts [1,32,40,41,65]. At the same time, conceptual ambiguity remains regarding what constitutes a mechanism [73,74]. By taking processes as its primary analytic focus, our narrative analysis offered complementary insights into how recovery unfolds in practice. These insights are less visible in mechanism-based descriptions. Theoretically, this suggests that, while mechanism-based explanations are helpful in articulating “why” interventions may facilitate conditions for change, these remain at a level of abstraction that obscures how recovery actually takes shape. Attempts to understand recovery may therefore also benefit from approaches that attend to “how” generative processes become meaningful, interact, and vary across persons and situations over time.

### Practical Implications

The 3 presented narratives offered situated insights into the value of experts-by-experiences’ (or peers’, more generally) unique “position to know” [76,77] through their bodily and emotional involvement in recovery processes [78-80]. This aligns with broader developments in mental health care and research, where experiential knowledge is increasingly recognized as an epistemically legitimate source alongside scientific and professional knowledge (eg, in discussions on epistemic justice [43]). This development is reflected in the growing professionalization of expertise-by-experience [81,82] and in care providers increasingly disclosing about personal lived experiences in their profession (eg, as “wounded healers” [66,83]). At the same time, the institutional embedding of expertise-by-experience is not without concerns, including risks of abstraction, cooptation, and assimilation within health care frameworks [76,82,84,85].

Within this context, the presented narratives illustrated how an RC (or other participatory health initiative) can explicitly facilitate space where experiential knowledge can be shared, explored, and developed. This dedicated space is characterized by organizational and relational conditions under which recovery could unfold (such as experiences of recognition, safety, and personalized opportunities to partake). Importantly, literature emphasizes that such “free space” does not emerge automatically and requires ongoing collective effort and explicit safeguarding within the community [33]. Also, our findings suggest that recovery as a learning process is not limited to cognitive or verbal exchange but also unfolds as an embodied process of doing [27,28]. This highlights the value of diverse participatory opportunities, including creative, embodied, and action-oriented practices, to support recovery as a practice-based learning process.

### Strengths and Limitations

The qualitative diary-interview design enabled situated insights into recovery processes. Incorporated in a large, mixed-methods research project, the study provides qualitative enrichment of quantitative RC evaluations [86,87] and a more elaborate, experiential description of the meaning of RC spaces [33] for recovery. A key strength lies in the study’s participatory and co-created design, which centered the research entirely on participants’ lived experiences. This approach particularly attended to power dynamics and meaningful participation for research participants, promoting ownership and agency [45,46]. Collaborative data collection and analysis contributed to ongoing reflexive dialogues, acquiring insights beyond pre-existent personal and/or academic beliefs [45].

The study also has limitations. Most importantly, the study is prone to selection bias in 2 ways. First, because diary studies demand considerable effort, participation is likely limited to RC partakers who have the capacity, resources, and motivation to sustain RC engagement and have a strong motivation to contribute to its evaluation. Second, participants indicated that they less frequently completed diaries on bad days. These forms of selection bias have also been cautioned against in literature [48,88]. Together, these dynamics may have contributed to the empirical material foregrounding experiences of growth while periods of stagnation or withdrawal are likely underrepresented. In addition, while the narratives captured diverse RC attendance in terms of duration and roles, the demographic diversity of the sample was limited. Furthermore, diary entries were guided by predefined themes, which may have limited the breadth of the narratives. Finally, participants could access the dashboard during the diary period, which might have stimulated additional in-between reflections and adjustments. However, dashboard usage was rare (limited to a single, curiosity-driven visit at the start), so its impact is likely negligible.

### Future Directions

We propose 2 directions for future research. First, RCs in the Netherlands centralize experiential knowledge and bottom-up co-creation by and for peers [34], while there are also RCs, for example, in the United Kingdom, that equally value and connect sources of professional, experiential, and scientific knowledge [24]. Building on our findings on the meaning of experiential knowledge in a context where it is the dominant source, future research could examine whether learning processes in contexts that combine multiple knowledge sources are comparable or distinct.

Second, research could further develop diary-based methods as participatory approaches by examining both the design of such methods and their potential effects on recovery processes. Regarding design, future studies could employ even more participatory designs by giving participants full agency in shaping the study (eg, in defining personal diary questions and collaborating during the entire analysis). Such approaches may better accommodate the uniqueness of recovery, advance ethical principles of ownership, and improve compliance, particularly during difficult periods. Additionally, complementing diaries with more immersive, context-sensitive methods (eg, home-based interviews or ethnographic methods) could help to situate diary entries within participants’ everyday contexts and support interpretation of what is recorded.

In terms of effects, literature suggests that journaling can deepen self-knowledge, increase awareness of patterns, and foster higher-order thinking, reflection, and meaning making [89]. In our study, such processes were most prominent in Robin’s narrative. While diary methods have been proposed as therapeutic tools with both positive and negative effects—predominantly evaluated in terms of symptom reduction (eg, [90,91])—their potential role in supporting personal recovery processes has received comparatively less attention. Future research could therefore examine how diary-based practices, particularly when designed in more participatory ways, influence the unfolding of recovery processes beyond clinical outcomes.

### Conclusions

Three narratives of RC partakers showed how their recovery unfolded as 2 learning processes: learning by exchanging and learning by doing. The RC facilitated space for both: reflecting upon experiences in exchange with peers, and experiencing, practicing, and doing new things. RC context was associated with supportive experiences of recognition, hope, and inspiration, reinforcing recovery processes to unfold. Partakers as such (re)built their self-confidence, empowerment, and a positive identity. Importantly, these processes did not progress in a linear fashion but were erratic: partakers also experienced challenges in their everyday lives and within the RC specifically. The narratives illustrated how RCs can be valuable assets in landscapes of mental care and support. Likewise, experiential knowledge was identified as a valuable knowledge source to learn from in recovery.

## Supplementary material

10.2196/89886 Multimedia Appendix 1 Norah’s animated narrative.

## Abbreviations

CHIME: connectedness, hope, identity, meaningfulness, and empowerment
HOP: Honest, Open, Proud
RC: recovery college
UWV: Uitvoeringsinstituut werknemersverzekeringen (Dutch Employee Insurance Agency)
VERS: Vaardigheden emotieregulatiestoornissen (skills emotion regulation disorders)
WRAP: Wellness Recovery Action Planning
